# Fatal infection caused by a genetically distinct elephant endotheliotropic herpesvirus type 5 in a captive Asian elephant in Germany

**DOI:** 10.1186/s12985-024-02477-w

**Published:** 2024-09-16

**Authors:** Azza Abdelgawad, Mariana Nascimento, Adriane Prahl, Michael Flügger, Claudia A. Szentiks, Susanne Holtze, Thomas B. Hildebrandt, Jakob Trimpert

**Affiliations:** 1 Institut für Virologie, Freie Universität Berlin, Robert-von-Ostertag-Str. 7–13, 14163 Berlin, Germany; 2 Tierpark Hagenbeck Gem. GmbH, Lokstedter Grenzstraße 2, 22527 Hamburg, Germany; 3 Leibniz-Institute for Zoo and Wildlife Research, Alfred-Kowalke-Strasse 17, 10315 Berlin, Germany; 4 Department of Diagnostic Medicine and Pathobiology, College of Veterinary Medicine, Kansas State University, Manhattan, KS USA

**Keywords:** EEHV5, Elephant, Full genome sequencing, SNPs, Haemorrhagic disease

## Abstract

**Background:**

Elephant endotheliotropic herpesvirus (EEHV) infection is the most common cause for lethal hemorrhagic disease in captive juvenile Asian elephants (*Elephas maximus*). Although EEHV1 is known as the most likely cause of fatal haemorrhagic disease in Asian elephants, EEHV5 was lately involved in lethal cases of haemorrhagic disease in captive elephants.

**Case presentation:**

Here we report the first death of a four-year old Asian elephant diagnosed with EEHV5 in Germany. Molecular diagnosis yielded detection of EEHV5 DNA in all tested tissues. Histopathological examination revealed typical features of hemorrhagic disease in all examined organs. EEHV5 was sequenced from total DNA isolated from heart tissue by Illumina and Nanopore sequencing. Sequencing data showed 3,881 variants, distributed across the entire genome, compared to the published EEHV5 sequence.

**Conclusions:**

We have detected EEHV5 in a fatal disease case of a male Asian elephant. Whole genome sequencing revealed substantial differences of our DNA isolate compared to available EEHV5 sequences. This report of fatal haemorrhagic disease associated with EEHV5 infection should raise awareness for EEHV5 as an important elephant pathogen. Genome sequencing and downstream SNPs analysis will further encourage future research to understand genetic diversity, pathogenesis and virulence of EEHVs with respect to developing new diagnostic methods, prophylactic strategies, and implementation of surveillance and control measures.

**Supplementary Information:**

The online version contains supplementary material available at 10.1186/s12985-024-02477-w.

## Background

Elephant endotheliotropic herpesviruses (EEHVs) are members of the *Orthoherpesviridae* family and belong to the genus *Proboscivirus* [1, 2]. Seven genotypes and five subtypes of EEHVs have been determined: EEHV1A and 1B, EEHV2, EEHV3A and 3B, EEHV4A and -4B, EEHV5A and 5B, EEHV6 and EEHV7A and 7B [3, 4]. Infection of elephants with all EEHV genotypes has been reported. These viruses pose a significant threat to both Asian (*Elephas maximus*) and African (*Loxodonta africana*) elephants causing haemorrhagic disease particularly in the young Asian elephants worldwide [5–7].

EEHV5 was first detected in a 59-year-old wild-born female Asian elephant in 2007 without signs of serious disease [2, 8]. In 2011, a 42-yr-old wild-born female Asian elephant that presented with bilaterally swollen temporal glands, oral mucosal hyperaemia, vesicles on the tongue and generalized lethargy was diagnosed with EEHV5 infection [8]. One of the two available EEHV5 whole genome sequences, “NAP58 Tucker” was obtained from a surviving asymptomatic infection of an 8-year old captive-born male Asian elephant in Texas [3]. The first fatality associated with EEHV5 was described in 2011. A 20-month-old Asian elephant, Vijay, was born on August 6th, 2009 at Twycross Zoo, UK and was euthanized following 6 days of unrecovered lethargy, edema, hemorrhages in different mucosal membranes and lingual cyanosis. Post-mortem examination of Vijay revealed symptoms characteristic of EEHV infection, including petechial to ecchymotic hemorrhages in different organs (conjunctiva, brain, esophagus, trachea, heart, stomach, intestine, liver, urinary bladder, and adrenal glands). This case yielded the other thus far available EEHV5 whole genome sequence [9]. One more EEHV5 case was reported in a captive elephant in China, which was diagnosed during a prevalence survey on EEHVs in captive animals and wild-life in China [10].

Diagnosis of EEHV-infection is mainly based on clinical signs in consideration of the elephants age, detection of viral nucleic acids in blood samples and/or trunk washes using quantitative PCR (qPCR), post-mortem gross lesions, histopathology, immunohistochemistry, and whole genome sequencing [7, 9, 11].

Here, we report full genome sequencing and the post-mortem characteristics of the first fatality associated with EEHV5 infection in a 4-years-old male captive Asian elephant in Germany.

### Case presentation

The sudden death of a 4-year old male Asian elephant “Raj” at Tierpark Hagenbeck, Hamburg, Germany was reported on June 9th, 2022. The elephant was captive bred and part of a heard comprised of 7 adult females and a young bull born in December 2018. Both bulls were born in Tierpark Hagenbeck, and were both parent-raised and part of an intact herd. At the time of illness, Raj was still together with his mother and was seen suckling at times. In the first three days of illness, Raj showed symptoms of lethargy and significant increased body temperature of 39 °C. Raj was treated with 15 mg/kg Famciclovir (FamVir® 500 mg Filmtabletten, Novartis) PO TID and 2.5 mg/kg enrofloxacin (Baytril 10%, Bayer) IM SID as recommended by the zoo veterinarian and was tested with all co-housed elephants for EEHV1 infection. One day before death, Raj showed cyanotic tongue and the blood analysis revealed a severe leucopenia (4,2 G/l) with lymphopenia (675 cells/µl) and thrombocytopenia (99 G/l). Additional treatment of 6 × 18 Mio. I.U. Interferon alpha-2b (Intron® A, MSD) SQ and 0.5 mg/kg Meloxicam (Emdocam® 20 mg/ml, WDT) IM was started immediately. It is worthy to mention that Raj had no previous history of EEHVs illness although two young elephants of his herd died from EEHV-1A in June 2018 when he was only few weeks old [7]. EEHV-5 infection was previously never reported in any member of the herd. After the death of Raj, as a precautionary measure, blood samples and trunk washes were collected from the other apparently-healthy in-contact elephants and tested for all EEHVs infections.

The carcass of “Raj” underwent complete necropsy by colleagues of Tierpark Hagenbeck and Leibniz Institute for Zoo and Wildlife Research, Berlin, Germany, where complete necropsy was performed and tissue samples were collected.

Tissues from lung, liver, heart, spleen, kidney, and lung-lymph nodes and blood samples were collected and shipped to the Institut für Virologie, Freie Universität Berlin for virological investigations. Viral DNA from tissue homogenates and blood was extracted using innuPREP virus DNA/RNA kit, (IST Innuscreen GmbH, Berlin, Germany) and innuPREP blood DNA Mini kit (Analytik Jena., Überlingen, Germany), respectively. All tissue samples were tested for all EEHVs; only EEHV5 was positive. The qPCR was performed as previously described [2, 7, 12]. Primers and probes specific for the EEHV5 DNA polymerase gene (Additional Table 1) were used. Viral genome copy numbers were normalized to cellular genome copies detected in the tested tissue samples with a primer/probe set (Additional Table 1) specific to elephant tumor necrosis factor (TNFα), exemplary qPCR products are visualized on an agarose gel (Additional Fig. 1). Viral DNA copy numbers were expressed as copies per million cells, we mathematically corrected for the fact that each diploid eukaryotic cell has two copies of the TNFα gene. The viral genome copies in blood were calculated per milliliter (ml) of whole blood.

**Fig. 1:**
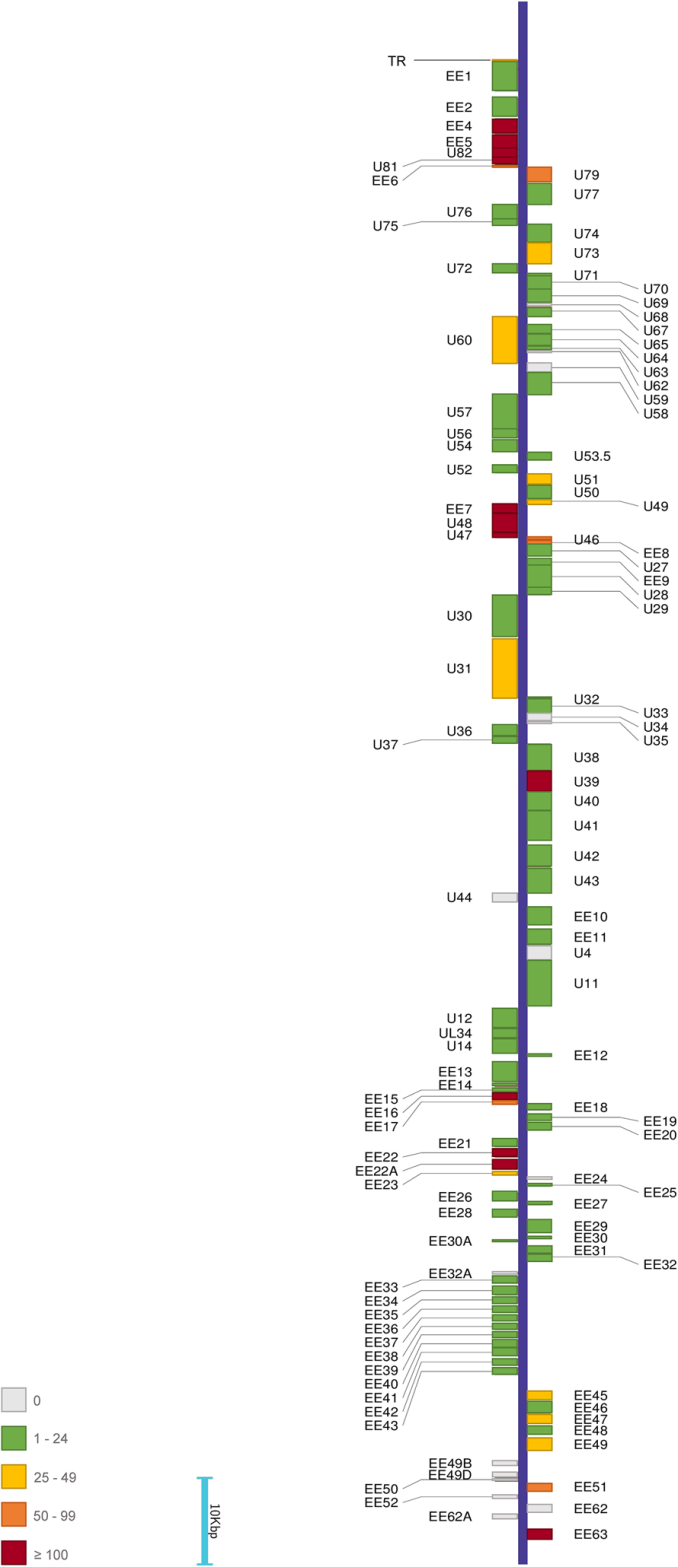
Representation of variant distribution across the EEHV5A Vijay genomic backbone. Color scale on the left illustrates the number of variants present in each gene according to gene color in the map; light blue bar represents the scale, using 10,000 bp (10 Kbp) as a unit. The initial EEHV5A Vijay genome map was drawn using GeneCo and modified to include variant information

For whole-genome DNA sequencing: DNA was extracted from heart tissue using the innuPREP Virus DNA/RNA Kit (IST Innuscreen GmbH, Berlin, Germany). High Molecular Weight DNA was extracted using the Circulomics Nanobind Tissue Big DNA Kit (https://www.circulomics.com/nanobind). For the latter, 3 parallel DNA extractions from 50 mg of tissue each; extractions were pooled at the end by eluting all nanobind disks in the same eluate.

DNA from heart tissue was sequenced using both short-read Illumina sequencing as well as long-read Nanopore sequencing. Illumina library preparation was conducted as described before using both innuPREP and Circulomics extracted DNA [7]. To fill sequence gaps encountered after Illumina sequencing, Oxford Nanopore sequencing runs were conducted using HMW DNA. Libraries were prepared using the Oxford Nanopore Ligation Sequencing kit (SQK-LSK110) and then sequenced on a FLO-MIN106 flow cell and a MinION MK-1B device. Library preparation was conducted according to the manufacturer’s protocol, with the exception of end-prep (both steps) and ligation incubation times, which were extended to 30’ each (https://community.nanoporetech.com/protocols/genomic-dna-by-ligation-sqk-lsk110/checklist_example.pdf?device=minion). The generated Illumina short-read sequencing data were processed with Trimmomatic v.0.39 [13] and mapped against either the sequence of previously published EEHV5 isolate “Vijay” [9] (GenBank accession number: KF921519.1) or the deposited EEHV5B isolate “North American NAP58 Tucker” sequence (GenBank accession number: OL982749.1) with the Burrows-Wheeler aligner v.0.7.17 [14]. Samtools v1.10 [15] was used to generate mapping statistics and IGV v2.9.4 for Linux [16] was used for alignment visualization and inspection. For detection of single-nucleotide polymorphisms (SNPs), Freebayes, a Bayesian genetic variant detector [17] was used with the following parameters: only SNPs with a minimum mapping quality of 5, minimum count of 3 and minimum fraction of 0.01 were considered. Consensus sequences were then obtained using BCFtools [14]. The pre-processing, mapping and variant calling steps are wrapped in a custom script (available at https://github.com/mmnascimento/EEHV5). As for Nanopore long-read data, basecalling was performed using the High Accuracy model, filtering out reads with less than 200 nt in length and/or reads with a Quality Score inferior to 8. Mapping was performed against either EEHV5A isolate Vijay (GenBank accession number: KF921519.1) or EEHV5B isolate North American NAP58 Tucker (GenBank accession number: OL982749.1) and, along with variant calling, was conducted using the medaka_haploid_variant workflow of Medaka v1.7.2 (https://github.com/nanoporetech/medaka). Consensus sequences were obtained using the medaka_consensus module of the same tool.Raw Illumina and Nanopore sequencing data, as well as alignment files for both datasets against each reference are available at the Sequencing Read Archive (SRA) database under BioProject number PRJNA1102414.Variant calling results from Illumina and Nanopore data were manually parsed and indels, along with any variants with coverage < 10 reads, were removed. Filtered results were then combined: variants detected using both technologies were included in final variant tables, as were variants detected solely via short-read sequencing; Nanopore long-read exclusive variants were subjected to further manual parsing, to assess concordance between short and long-read sequencing data. Nanopore-exclusive variants were detected in areas of the genome where Illumina coverage was either very low or non-inexistent. As such, long-read exclusive variant calls in low coverage areas were validated by direct inspection of the short-read data alignment using IGV v2.9.4 for Linux: for each Nanopore-exclusive variant, Illumina short reads in the corresponding genomic position were investigated: for both references, Illumina data mostly agreed with Nanopore data, but low coverage did not allow for a formal call with Illumina short read data alone. At this point in the analysis, all variants where short-read data coverage is > 10 reads and the long-read data directly contradicts short-read information were eliminated. Given the very high concordance rate between short and long reads, long-read exclusive variants in regions with low or non-existent short-read coverage were also included in final variant tables. Two final consensus sequences reflecting the curated selection of variants on each of the references used for mapping were then created using bcftools consensus [15]. These final consensus sequences are available in the aforementioned repository (https://github.com/mmnascimento/EEHV5) as well as via GenBank: the consensus in EEHV5A background is available under accession number PP906086 and the consensus in EEHV5B background is available under accession number PP906087. Genome maps of EEHV5A isolate Vijay and or EEHV5B isolate North American NAP58 Tucker were drawn using GeneCo [18] and modified to include variant information, representing the EEHV5A and EEHV5B consensus, respectively (Fig. 1, Sup Fig. 1).

To assess the phylogenetic placement of the references used and newly obtained consensus sequences, several phylogenetic trees previously generated for comparative genome analysis of EEHV3, EEHV4, EEHV5 and EEHV6 were recreated here. In addition to the original sequences, corresponding regions from EEHV5A isolate Vijay, EEHV5B isolate North American NAP58 Tucker and our obtained consensus sequences were included.

An intra-EEHV radial phylogenetic tree was constructed, based on U38 (POL, DNA polymerase), and inferred using the Neighbor-Joining method [19]. The evolutionary distances were computed using the Maximum Composite Likelihood method [20]. Ambiguous positions were removed for each sequence pair, leaving 1075 positions in the final dataset. Traditional DNA level phylogenetic trees (Fig. 3) were created for U38 (POL, DNA polymerase) and U73 (OBP, origin-binding protein), comparing EEHV members with relevant herpesvirus representatives. Both trees were built using the Maximum Likelihood Method. The evolutionary history of both trees was inferred by using the Maximum Likelihood method and Jukes-Cantor model [21] on final datasets of 1075 positions for U38 (POL) and 672 positions for U73 (OBP). The bootstrap consensus tree inferred from 500 replicates is taken as representative of the evolutionary history of the taxa analyzed. The percentage of replicate trees in which the associated taxa clustered together in the bootstrap test (500 replicates) are shown next to the branches [22].

A traditional protein level phylogenetic tree (Fig. 4) was also generated for U81 (UDG, uracil-DNA glycosylase), comparing EEHV members with relevant herpesvirus representatives. This tree was built using the Maximum Likelihood method and JTT matrix-based model [22, 23] on a final dataset of 257 positions. All phylogenetic trees were built using MEGA 11 [24, 25]. Where protein translation and pairwise alignments were required for both construction and interpretation of the phylogenetic trees, proteins were translated using the Expasy translate tool and aligned using the Expasy SIM tool [26]. Protein alignment and phylogenetic tree files, as well as any relevant intermediate files are also available in the Github repository (https://github.com/mmnascimento/EEHV5). Further methodological details and specific accession numbers can be found in the supplementary materials of the original publication [3].

Finally, we attempted virus isolation using Crandell-Rees Feline Kidney Cell (CrFK) and Elephant fibroblast (ENL-2) as well as fibroblasts extracted from Raj skin cell cultures as described previously [7].

## Results

Gross post-mortem examination revealed severe vascular lesions in all organs with extensive haemorrhages in heart, skeletal muscles, small and large intestines, and lung. The heart showed acute petechial to ecchymal hemorrhage in the subepicardial, intracardiac and subendocardial region with edema in the area of adipose tissue. The spleen had acute subcapsular petechial hemorrhages. The lymph nodes were hemorrhagic, diffuse and enlarged, especially intestinal lymph node. The liver showed extensive subcapsular edema with hemorrhagic rounded edges. The tongue was cyanotic and tinged with blue Esophagus. The small intestine presented with hemorrhagic walls with edema. On the skin multifocal petechial hemorrhage in the subcutaneous tissue was observed (Additional Fig. 2). Bacteriological examination of the liver and bronchial mucus revealed the presence of gram-positive cocci after enrichment and *Streptococcus alactolyticus*, respectively.

Histological findings were widely similar to the cases previously assigned to other EEHV genotypes, mainly characterized by haemorrhages, oedema and congestion. Inflammation was nearly absent, and despite intensive investigation, inclusion bodies, which are characteristic for other herpesvirus infections, were not detected.

Molecular analysis on the extracted DNA confirmed the presence of EEHV5 DNA in all tested tissues. Viral genome copies were quantified in infected tissues (Table 1). Attempts to isolate the virus from infected tissues as described before [7] were unsuccessful.

**Table 1:** Viral DNA copies assayed for the EEHV5 DNA polymerase gene and normalized to cellular DNA in tissues and per ml blood by qPCR

Specimen	Viral DNA copies
Lung	1.57 × 10^6^/million cells
Liver	3.85 × 10^6^/million cells
Heart	4.24 × 10^8^/million cells
Spleen	2.07 × 10^6^/million cells
Kidney	9.98 × 10^4^/million cells
L.node	3.29 × 10^5^/million cells
Blood	5.1 × 10^3^/ml

We sequenced the viral genome using DNA extracted from the elephant’s heart where viral DNA was most abundant. Illumina short-read sequencing was employed to determine possible variants and Oxford Nanopore long-read sequencing was used to complement Illumina data and fill in sequence gaps and low-coverage regions. Given that no protocols were available for EEHV5 enrichment, whole-genome sequencing was performed for both technologies, resulting in data containing both viral and host information. To minimize interference from host data, analysis was geared towards a reference-based approach and larger complex structural variants were discarded. As previous molecular analysis placed this isolate within the EEHV5 genotype, obtained sequencing data was first mapped against the only peer-reviewed complete EEHV5 genome: EEHV5A isolate Vijay (henceforth referred to as EEHV5A Vijay). Since the analytical focus was on detection of SNPs and short-spanning nucleotide substitutions, the genomic structure of the generated consensus corresponds directly to the reference’s structure.

The data in Additional Table 2 shows variants obtained after analysis of the entirety of our sequencing data. Interestingly, the obtained consensus was considerably different from the previously published genome. Using EEHV5 Vijay as a reference we determined 3,881 variants; 444 of those are located outside open reading frames (ORFs) and 45 are located within terminal repeats (TR). Of the 3,881 detected variants, 972 were found in both short-read and long-read data, 393 were only supported by short-read data and 2,516 were only supported by long-read data; within long-read only variants, 620 were supported by manually parsing the mapping of short reads and 1896 were located in regions that short-read data did not cover. A representation of the detected variation across the Vijay reference can be found in Fig. 1. Variants are generally distributed throughout the genome; however, some genes do not harbor any (Additional Table 2).

**Fig. 2:**
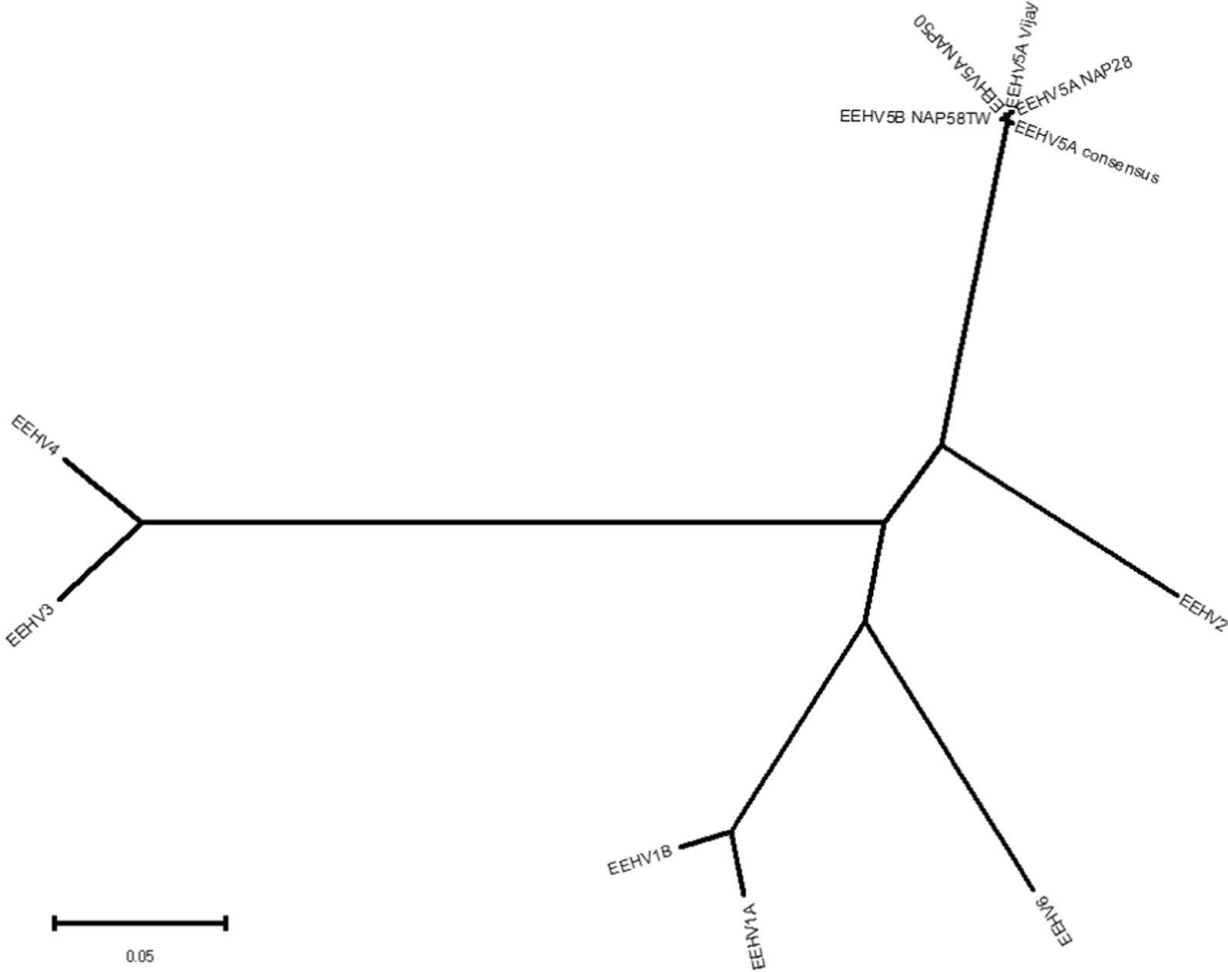
DNA level evolutionary relationships between the obtained EEHV5A consensus sequence and EEHV family representatives. The radial phylogenetic tree is based on the U38 (POL) gene and was inferred using the Neighbor-Joining method. All codon positions were included. Ambiguous positions were removed for each sequence pair, leaving 1075 positions in the final dataset. The bar displays the number of nucleotide substitutions per site. Evolutionary analysis was conducted using MEGA11

**Fig. 3:**
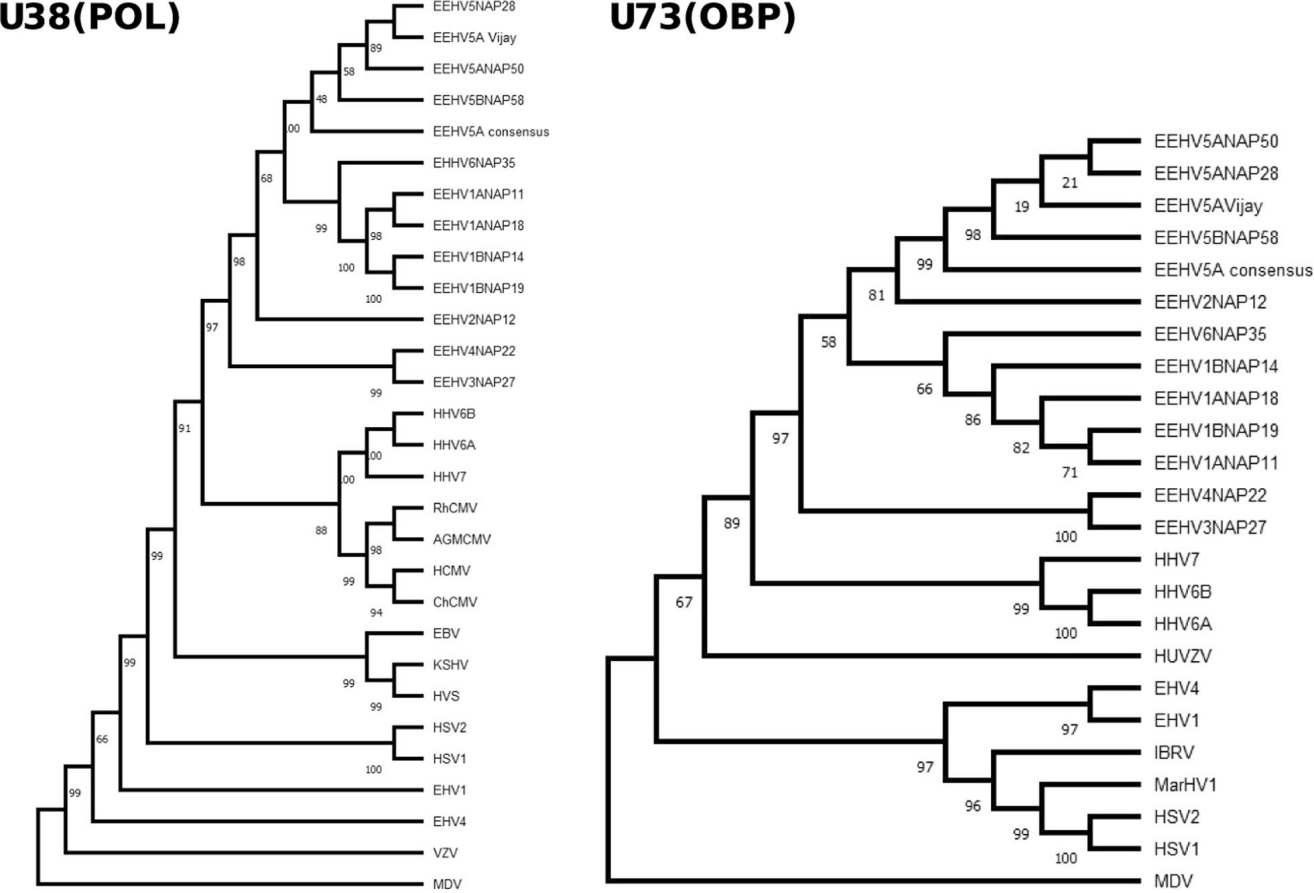
DNA level evolutionary relationships between the EEHV5A consensus, EEHV family representatives and other relevant herpesviruses. The linear phylogenetic trees are based on U38 (POL) and U73 (OBP) genes, respectively. Trees were inferred using the Maximum Likelihood method and Jukes-Cantor model. All codon positions were included and ambiguous positions were removed for each sequence pair, leaving 1075 positions in the final dataset for U38 (POL) and 672 positions in the final dataset for U73 (OBP). The percentage of replicate trees in which the associated taxa clustered together in the bootstrap test (500 replicates) are shown next to the branches. Evolutionary analysis was conducted using MEGA11

**Fig. 4:**
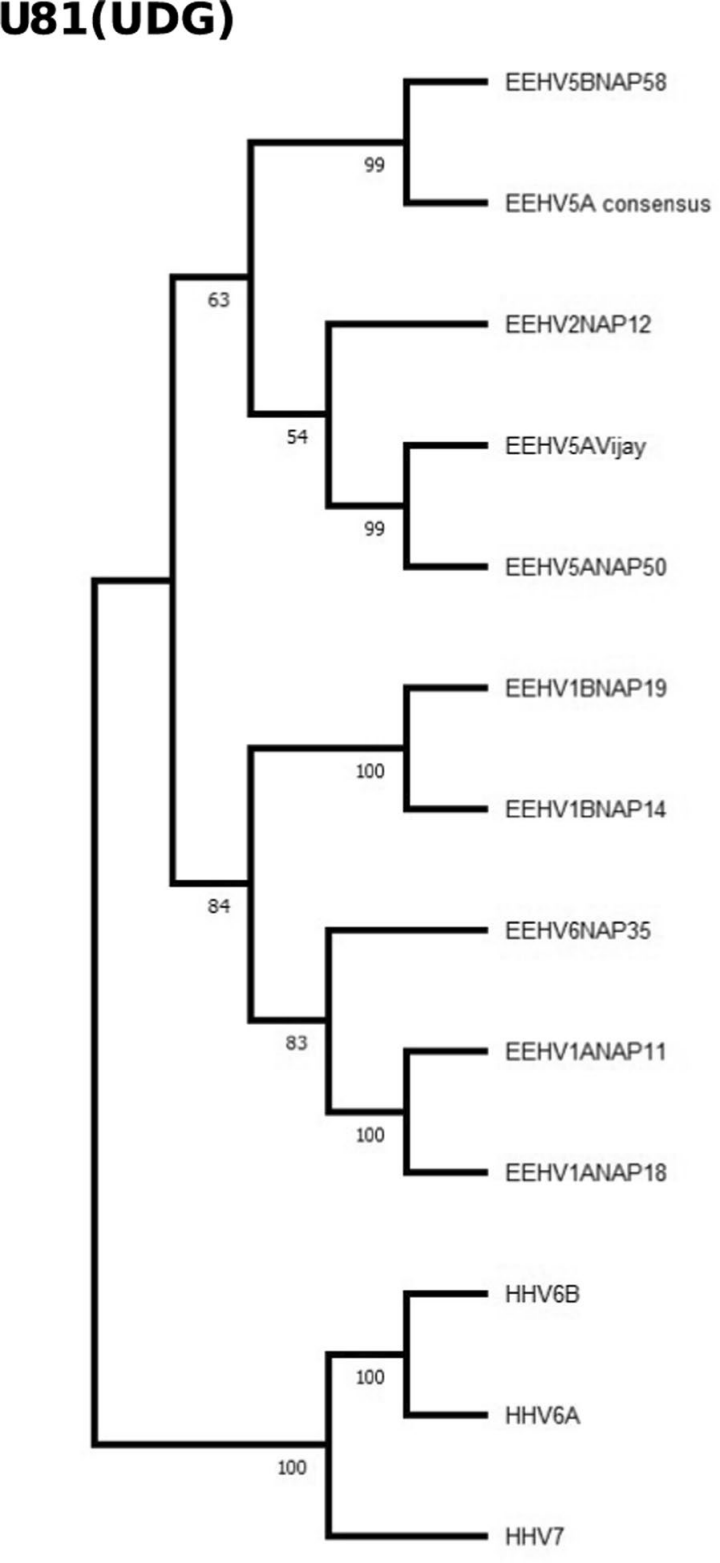
Protein level evolutionary relationships between the EEHV5A consensus, EEHV members and Betaherpesvirinae subfamily representatives. The radial phylogenetic tree is based on the U81 (UDG) protein sequence and was inferred using the Maximum Likelihood method and JTT matrix-based model. The final datased included 257 positions. The percentage of trees in which the associated taxa clustered together is shown below the branches. Full evolutionary analysis was conducted using MEGA11

To gauge the positioning of our data, several phylogenetic trees previously used for placement of EEHV5 isolates in relation to other EEHVs and herpesviruses were recreated (Figs. 2, 3 and 4) [3]. The original publication relied on partial sequences, mostly obtained via Sanger sequencing and to these, the corresponding regions from the EEHV5A Vijay genome as well as from our consensus sequence (factoring in all detected variants) were added. Three DNA-level phylogenetic trees were constructed: two of the trees are based on U38 (DNA polymerase), for placement within the EEHV group only and for comparison with other key herpesviruses, respectively; the last tree is based on U73 (origin-binding protein), also used for comparison of EEHVs with key herpesviruses. Additionally, one protein-level tree was constructed using U81 (uracil DNA glycosylase) and encompassing key herpesviruses. The selected loci and protein segments contain different levels of variation between our data and the references, and contain enough resolution to allow differentiation between EEHV5 isolates.

The intra EEHV5 Polymerase (U38) radial tree (Fig. 2) very closely replicates the original. It places the Vijay reference and the consensus with the remaining EEHV5 isolates, as expected. When extending the analysis to include key herpesviruses (Fig. 3), EEHV5A Vijay clusters along with EEHV5A NAP28. Despite our data containing only 18 variants in relation to EEHV5A Vijay, these are sufficient for the consensus to cluster within an EEHV5 group but peripherally from all other isolates. However, when translating the nucleotide consensus sequence into an amino acid sequence there are no differences detected, which argues for high functional conservation of the DNA polymerase as observed across many herpesviruses.

The remaining DNA-level tree, based on the Origin-Binding Protein (U73) (Fig. 3), also places the EEHV5 sequences closely, with EEHV5A Vijay clustering outside the EEHV5A NAP50 and NAP28 branch. Similarly to the U38 tree, EEHV5A consensus is placed peripherally to the remaining EEHV5 isolates. Our data’s U73 sequence contains 25 variants in relation to EEHV5A Vijay, that translate into 4 differences at the amino acid level.

Lastly, the protein-based phylogenetic tree was built using the amino acid sequence of the Uracil DNA Glycosylase (U81) (Fig. 4) and it clusters EEHV5A Vijay along with EEHV5A NAP50 as observed in the original tree. The newly generated consensus is again in a different branch, slightly closer to EEHV5B isolates. In the case of U81, the number of variants relative to the reference is quite high at 173 detected variants.

Overall, the consensus is placed close to the remaining EEHV5 isolates, but in distinct branches from the reference, with the structure of each tree reflecting that of the original trees [3]. Variant and phylogenetic analysis suggest that our data is different from the EEHV5A Vijay genome. At this point, no assertions could be made about how the data relates to EEHV5B genomes.

To assess this, we used a GenBank entry corresponding to a full EEHV5B genome: EEHV5B isolate North American NAP58 Tucker (henceforth referred to as EEHV5B Tucker). However, the manuscript associated with this genome is not yet published, making it a non-peer reviewed entry. Due to the scarcity of genomic information for EEHV5 viruses, we nevertheless decided to use it as a secondary reference. All previous analytical steps were repeated using EEHV5B Tucker as a reference: variant calling was performed and a new consensus sequence was derived (EEHV5B consensus); all phylogenetic analyses were also repeated to include EEHV5B Tucker and the new EEHV5B consensus. As depicted in Additional Fig. 3, genome size differs slightly between EEHV5A Vijay and EEHV5B Tucker—the EEHV5A Vijay genome is 5 kbp longer than EEHV5B Tucker; additionally, there is also substantial inversion between the two, spanning a 103 kbp region. This region, albeit inverted, harbours a consistent and common repertoire between the two genomes, containing most genes from U27 to U82. However, each isolate encodes a few unique proteins scattered across the genome related to gene regulation, as well structural proteins involved in capsid and tegument structuring.

Using EEHV5B Tucker as a reference we determined 3,083 variants (Additional Table 3); 160 of those are located outside ORFs and 24 are located within terminal repeats. Of the 3,083 detected variants, 1258 were detected with both short and long-reads, 14 were only supported by short-read data and 1550 were only supported by long-read data; within these variants, 171 were supported by manually parsing the mapping of short reads and 1379 were located in regions that short-read data did not cover. A representation of the detected variation across the Tucker reference can be found in Additional Fig. 4. For EEHV5B variants are also distributed throughout the genome, with the exception of a few genes that contain no variants.

To gauge the positioning of our data in light of EEHV5B, all previously mentioned trees (Figs. 2, 3 and 4) were re-built with the addition of EEHV5B Tucker and the EEHV5B consensus and can be found in Additional Figs. 5, 6 and 7. The intra EEHV5 Polymerase (U38) radial phylogenetic tree (Additional Fig. 5) continues to very closely replicate the structure of the original tree. As for the U38 tree including other herpesviruses (Additional Fig. 6) EEHV5B Tucker clusters along with EEHV5B NAP58. EEHV5B Tucker and EEHV5B NAP58 are anticipated to cluster together, given that the Tucker genome reference is also labelled as NAP58TW: there is a difference of one amino acid between the two protein sequences, which might be due to the methodology used to obtain the full Tucker genome. Levels of variation for this protein are similar with 18 and 19 SNPs in relation to EEHV5A Vijay and EEHV5B Tucker, respectively.

The Origin-Binding Protein (U73) phylogenetic tree (Additional Fig. 6) places all the EEHV5 sequences closely once more, with EEHV5A Vijay clustering outside the EEHV5A NAP50 and NAP28 branch, followed by EEHV5B NAP58 and finally EEHV-5B Tucker. Although belonging to a similar isolate, the EEHV5B NAP58 sequence is incomplete in comparison with the EEHV5B Tucker genome. Similarly to the U38 based tree, EEHV5A and EEHV5B consensuses are placed peripherally to other EEHV5 isolates and cluster together. Our data’s U73 sequence contains 25 and 24 variants in relation to the EEHV5A Vijay and EEHV5B Tucker respectively, translating into 4 and 2 differences at the amino acid level.

Lastly, the Uracil DNA Glycosylase (U81) tree (Additional Fig. 7) clusters EEHV5B Tucker along with EEHV5B NAP58 and otherwise resembles the original tree and the tree in Fig. 4. Also for this protein, one difference at the amino acid level between EEHV5B NAP58 and EEHV5B Tucker can be seen. The newly generated consensuses are again in different branches, placed slightly closer to EEHV5B isolates. For U81, the number of variants relative to each reference is quite different: for EEHV5A Vijay our data contains 173 variants, whilst for EEHV5B Tucker there are only 3. These translate onto 34 and 1 changing amino acids, indicating that this protein is more similar to the EEHV5B sequence.

In all phylogenetic trees containing EEHV5B Tucker and the EEHV5B consensus, both consensuses are placed close to EEHV5 isolates, but in distinct branches, usually clustering together and indicating that our data is different from the available EEHV5A and B genomes.

### Discussion and conclusions

EEHVs continue to be one of the main threats to elephants in captivity. EEHV1A and B are long known to be a major main cause of fatal disease in young (Asian) elephants in captivity, with many cases described in the last three decades [5, 6]. EEHV5, on the other hand, has been associated with non-fatal clinical infection only until the first fatal case was reported in 2011 in the UK. EEHV infections are considered ubiquitous among elephant populations in captivity and in free range [3, 27, 28], however fatal incidences of haemorrhagic disease in young elephants remain peculiar as they seem to contradict the smooth coexistence of virus and host achieved through long standing co-evolution [29].

Here, we report another fatal case associated with EEHV5 in a young Asian elephant (and the first case in continental Europe). Clinical signs appeared for a brief period before leaving little room for diagnostic measures and therapeutic intervention. Haemorrhagic lesions were observed in most of the visceral organs and tissues, which is typical for fatal cases related to EEHV infection [4, 5, 7, 9, 11, 28]. Although typical for herpesvirus infection, we did not observe intranuclear inclusion bodies despite extensive histological search. We detected EEHV5 DNA in various tissues throughout the elephants body, which again resembles findings from previous EEHV associated fatalities [7, 9, 29]. This pan-body tropism of the virus might be attributed to its endotheliotropic nature with subsequent extensive replication, in the endothelial lining of all tissues. Since EEHV5 infection was thus far not associated to severe disease in captive elephants in continental Europe, standard testing protocols for sick Asian elephants do not necessarily include EEHV5 testing. We hope that this report will raise awareness for the potentially fatal nature of EEHV5 infection in Asian elephants and strongly encourage inclusion of EEHV5 in virological diagnostics for this species.

We further continued our efforts and have attempted to isolate EEHV5 on CrFK (feline), ENL-2 (elephant) and elephant fibroblasts (extracted from Raj skin) cell cultures. Unfortunately, our attempts remained unsuccessful: after the 2nd virus passage on each cell line, no viral DNA could be detected from cultured cells. As previously reported [6, 7], virus replication was not supported in cell culture. The reason for the inability to culture EEHVs remains elusive, we speculate that this may be due to the lack of suitable cultured host cells or missing of tissue complexity required for virus replication as provided by the natural host. This inability to support viral replication in cell culture reverberates into the amount of genomic information available for EEHV5 viruses, as there is a lack of robust references and genomic sequences which, in turn, complicates both the analysis of new sequencing data and the establishment of molecular enrichment methods for these viruses. Currently, no wet-lab EEHV enrichment or host depletion methods are available, meaning that EEHV-targeted sequencing is not yet a possibility. With cell culture also being unavailable, only whole-sample sequencing from the tissue of an infected animal is possible. This technique, which was employed in this study, carries a high percentage of host DNA that cannot be separated in wet-lab procedures. In such conditions, no analytical route is without disadvantages, making genomic analysis rather delicate. Although in silico host depletion is an option, it also may hinder the recovery of parts of the viral genome that may closely resemble that of the host; as homology between herpesviruses and their hosts is well documented and host mimicry is a relevant part of the viral evolutionary strategy, these regions may prove relevant to recover [30, 31]. While opting for reference-based approaches and employing stricter mapping parameters, as well as focusing on SNPs and small substitutions, is helpful in minimizing possible “background noise” from host DNA without loss of information on these common genomic areas, it does preclude detection of larger structural variation and structural novelty within the data that can only be found using de novo assemblies. Due to their stringency, mapping-based strategies may then overestimate the similarity between the sequencing data and the references used, undermining potential structural differences. Using a mapping approach on long and short-read next-generation sequencing derived from heart tissue, we could nevertheless determine variation across both the whole 180 kb genome sequence of EEHV5A Vijay, the only published and peer-reviewed reference available to us. Bioinformatic analysis of data obtained in the current study confirms the relatedness of the virus to EEHV5 isolates, but also reveals significant differences between the reference and our consensus sequence: almost 4000 variants were detected all over the genome relative to EEHV5A Vijay. One single gene (glycoprotein H, gH) showed alone 400 SNPs that result in 212 amino acid changes to this protein relative to the reference. This gene is well known to be conserved among all herpesviruses and involved in virus entry and/or cell fusion [32]. The role of gH as well as all other genes of EEHVs is not yet studied. Albeit in lesser quantity, variants at the protein level were also discovered in other typically highly conserved structural genes: for the large tegument protein encoded by U31, 2 amino acid variants were detected; for the DNA packaging protein 2, encoded by U50, 2 amino acid variants were detected; for major capsid protein U57, 5 amino acid variants were detected; for package subunit 1 encoded by U64, 6 amino acid variants were detected; for the portal protein encoded by U76, 2 amino acid variants were detected; for the terminase subunit encoded by U60, amino acid changes involve major structural changes that result in only 388 out of 660 amino acid residues aligning with the reference protein, and within this segment 9 amino acid variants were detected. Our data is thus substantially different from the published EEHV5A genome, at the nucleotide and amino acid levels. We could not, however, discard the possibility that the present data might represent an isolate closer to EEHV5B than EEHV5A. So far, there is only one complete EEHV5B genome sequence available in GenBank, which, at the date of writing this report, is still undergoing submission in a peer-reviewed journal [9]. However, due to the shortage of data regarding EEHV5 whole genomes, with no other EEHV5B sequence available, EEHV5B Tucker was used as a secondary reference in this study, to complement the main body of results derived from using EEHV5A as a reference. Looking at this additional body of results, over 3,300 variants were detected relative to EEHV5B Tucker, highlighting the unique character of this data amongst EEHV5 isolates, regardless of subgroup. When using the obtained 5A and 5B consensus sequences to recreate previously established phylogenetic dendrograms, they are consistently clustered together and placed in the periphery of other EEHV5 isolates, for both DNA and protein-level phylogenies, across proteins with differing counts of variants detected. Along with the results qPCR EEHV testing and variant calling, phylogenetic analysis reveals differences that point to the presence of an EEHV5 isolate in this sample that is seemingly distinct from the EEHV5 genomes described so far, for both 5A and 5B subgroups.

This division of EEHV5 into A and B subgroups was created to highlight the chimeric nature of EEHV5A and 5B genomes, which contain hypervariable sequence patterns among several loci, likely indicating ancient chimeric exchange events between A and B [3]. This chimeric tendency of the viral genome is shared with EEHV1A and EEHV1B, a group subdivided thusly for the same reason, and known to be the most likely cause of fatal haemorrhagic disease in Asian elephants [3]. The genotype EEHV7, also divided into A and B subgroups presumably for the same reason, has recently been implicated in a case of fatal disease in an African elephant, the first involving EEHV7 [33]. Due to the limited number of available EEHV5A and 5B genomes, it is difficult to know the full extent of these possible chimeric exchanges and how ancient or recent they may be, as well as what effect they might have on the virulence of EEHV5 isolates and the severity of disease caused by them. Due to the involvement of EEHV5 isolates in fatal cases of disease, the possible emergence of previously unknown EEHV5 genomes should not be overlooked.

Almost all our knowledge about herpesvirus genes is gained from human herpesviruses and few animal herpesviruses considered to be important for livestock. Lack of funding greatly hinders basic research on animal and exotic herpesviruses and decelerates the development of useful genomic and cell culture tools to address these questions in full.

As such, we leave this available for the scientific community for any possible future work that will provide a basis for better understanding of epidemiology, host–pathogen interaction and evolution of EEHV5 virulence. One possible application of our analysis could be the development of novel SNP-based PCR assays that can allow the detection of EEHV5 strains and help accurately determine the incidence of the virus. The genomic information included here could also be used to design EEHV5 DNA enrichment protocols for re-sequencing and genome walking, eventually allowing for de novo assemblies and thus inference upon larger structural genomic differences that may be present and could not be unveiled using the current methodology.

In conclusion, we have detected and studied the pathology of EEHV5 in fatal case of an Asian elephant. We encourage inclusion of EEHV5 in virological diagnostics of Asian elephants, even if no previous reports of EEHV5 related death or illness are available from a certain region or country. We believe that the reported EEHV5 DNA genomic analysis and consensus sequences will help further to understand the pathogenesis and virulence of EEHVs with respect to developing new diagnostic methods, prophylactic strategies, and implementation of control measures in future.

## Supplementary Information

Additional Figure 1: EEHV5 qPCR products visualized by agarose electrophoresis and ethidium bromide staining. Lane 1: 1 kb DNA ladder Plus (Thermo), Lane 2: EEHV5 (Raj spleen), Lane 3: EEHV5 (Raj heart).

Additional Figure 2: Post-mortem examination of elephant endotheliotropic herpesvirus (EEHV5)-infected elephant. Severe subepicardial petechial hemorrhages and edema in the area of adipose tissue in heart (a). Petechial hemorrhages in serosal surface of large bowel (b) and in abdomen (c).

Additional Figure 3: Comparison between the EEHV5A isolate Vijay and EEHV5B isolate Tucker genome maps. Every ORF is indicated by a grey box; the light blue bar represents the scale, using 10,000 bp (10 Kbp) as a unit. The genome map comparison was drawn using GeneCo.

Additional Figure 4: Representation of variant distribution across the EEHV5B Tucker genomic backbone. Color scale on the left illustrates the number of variants present in each gene according to gene color in the map; light blue bar represents the scale, using 10,000 bp (10 Kbp) as a unit. The initial EEHV5B Tucker genome map was drawn using GeneCo and modified to include variant information.

Additional Figure 5: DNA level evolutionary relationships between the EEHV5A and EEHV5B consensus and EEHV family representatives. The radial phylogenetic tree is based on the U38 (POL) gene and was inferred using the Neighbor-Joining method. It includes the EEHV5B Tucker reference genome. All codon positions were included. Ambiguous positions were removed for each sequence pair, leaving 1075 positions in the final dataset. The bar displays the number of nucleotide substitutions per site. Evolutionary analysis was conducted using MEGA11.

Additional Figure 6: DNA level evolutionary relationships between the EEHV5A and EEHV5B consensus, EEHV family representatives and relevant herpesviruses. The linear phylogenetic trees are based on U38 (POL) and U73 (OBP) genes, respectively. Trees were inferred using the Maximum Likelihood method and Jukes-Cantor model. It includes the EEHV5B Tucker reference genome. All codon positions were included and ambiguous positions were removed for each sequence pair, leaving 1075 positions in the final dataset for U38 (POL) and 672 positions in the final dataset for U73 (OBP). The percentage of replicate trees in which the associated taxa clustered together in the bootstrap test (500 replicates) are shown next to the branches. Evolutionary analysis was conducted using MEGA11.

Additional Figure 7: Protein level evolutionary relationships between the EEHV5A and EEHV5B consensus, EEHV and Betaherpesvirinae subfamily representatives. The linear phylogenetic tree is based on the U81 (UDG) protein sequence and was inferred using the Maximum Likelihood method and JTT matrix-based model. It includes the EEHV5B Tucker reference genome. The final dataset included 257 positions. The percentage of trees in which the associated taxa clustered together is shown below the branches. Full evolutionary analysis was conducted using MEGA11.

Additional Table 1 (Additional_table_1.xlsx): List of EEHV5 and elephant (TNFα) specific primers, probes, and oligonucleotides used for qPCR.

Additional Table 2 (Additional_table_2.xlsx): Detected variants after mapping to EEHV5A Vijay. The table in sheet #1 contains all variants detected using Illumina data, Nanopore data or confirmed by both datasets after mapping to the EEHV5A Vijay reference genome. The table in sheet #2 contains all coding sequences present in the reference genome, as well as their functions and annotation notes, along with how many variants were detected in each coding sequence of the EEHV5A Vijay reference genome.

Additional Table 3 (Additional_table_3.xlsx): Detected variants after mapping to EEHV5B Tucker. This table in sheet #1 contains all variants detected using Illumina data, Nanopore data or confirmed by both datasets after mapping to the EEHV5B Tucker reference genome. The table in sheet #2 contains all coding sequences present in the reference genome, as well as their functions and annotation notes, along with how many variants were detected in each coding sequence of the EEHV5B Tucker reference genome.

## Data Availability

Raw sequencing data as well as alignments of all datasets against all references are available at the Sequencing Read Archive (SRA) database under BioProject number PRJNA1102414. Custom scripts and consensus sequences are available at a dedicated Github repository: https://github.com/mmnascimento/EEHV5. The consesus EEHV5 genomes generated in this study are also availble via GenBank: the consensus in EEHV5A background isdeposited under accession number PP906086 and the consensus in EEHV5B background is available under accession number PP906087. All other data generated or analyzed during this study are included in this published article and its additional information files.

## Abbreviations

EEHV: Elephant endotheliotropic herpesvirus
TNFα: Tumor necrosis factor
qPCR: Quantitative polymerase chain reaction
SNPs: Single nucleotide polymorphism
ENL-2: Elephant fibroblast (Elephant cells)
CrFK: Crandell-Rees Feline Kidney Cell (Feline cells)

## References

[1] Gatherer D, Depledge DP, Hartley CA, Szpara ML, Vaz PK, Benko M, Brandt CR, Bryant NA, Dastjerdi A, Doszpoly A, et al. ICTV virus taxonomy profile: herpesviridae 2021. J Gen Virol. 2021;102:001673. 34704922 10.1099/jgv.0.001673 PMC8604186

[2] Latimer E, Zong JC, Heaggans SY, Richman LK, Hayward GS. Detection and evaluation of novel herpesviruses in routine and pathological samples from Asian and African elephants: identification of two new probosciviruses (EEHV5 and EEHV6) and two new gammaherpesviruses (EGHV3B and EGHV5). Vet Microbiol. 2011;147:28–41. 20579821 10.1016/j.vetmic.2010.05.042 PMC2976818

[3] Zong JC, Latimer EM, Long SY, Richman LK, Heaggans SY, Hayward GS. Comparative genome analysis of four elephant endotheliotropic herpesviruses, EEHV3, EEHV4, EEHV5, and EEHV6, from cases of hemorrhagic disease or viremia. J Virol. 2014;88:13547–69. 25231309 10.1128/JVI.01675-14 PMC4248975

[4] Long SY, Latimer EM, Hayward GS. Review of elephant endotheliotropic herpesviruses and acute hemorrhagic disease. ILAR J. 2016;56:283–96. 26912715 10.1093/ilar/ilv041 PMC4765743

[5] Richman LK, Montali RJ, Garber RL, Kennedy MA, Lehnhardt J, Hildebrandt T, Schmitt D, Hardy D, Alcendor DJ, Hayward GS. Novel endotheliotropic herpesviruses fatal for Asian and African elephants. Science. 1999;283:1171–6. 10024244 10.1126/science.283.5405.1171

[6] Ossent P, Guscetti F, Metzler AE, Lang EM, Rubel A, Hauser B. Acute and fatal herpesvirus infection in a young Asian elephant (Elephas maximus). Vet Pathol. 1990;27:131–3. 2161138 10.1177/030098589002700212

[7] Pavulraj S, Eschke K, Prahl A, Flugger M, Trimpert J, van den Doel PB, Andreotti S, Kaessmeyer S, Osterrieder N, Azab W. Fatal elephant endotheliotropic herpesvirus infection of two young Asian elephants. Microorganisms. 2019;7:396. 31561506 10.3390/microorganisms7100396 PMC6843339

[8] Atkins L, Zong JC, Tan J, Mejia A, Heaggans SY, Nofs SA, Stanton JJ, Flanagan JP, Howard L, Latimer E, et al. Elephant endotheliotropic herpesvirus 5, a newly recognized elephant herpesvirus associated with clinical and subclinical infections in captive Asian elephants (Elephas maximus). J Zoo Wildl Med. 2013;44:136–43. 23505714 10.1638/1042-7260-44.1.136 PMC3746547

[9] Wilkie GS, Davison AJ, Kerr K, Stidworthy MF, Redrobe S, Steinbach F, Dastjerdi A, Denk D. First fatality associated with elephant endotheliotropic herpesvirus 5 in an Asian elephant: pathological findings and complete viral genome sequence. Sci Rep. 2014;4:6299. 25199796 10.1038/srep06299 PMC5385831

[10] Yang N, Bao M, Zhu B, Shen Q, Guo X, Li W, Tang R, Zhu D, Tang Y, Phalen DN, Zhang L. Elephant endotheliotropic herpesvirus 1, 4 and 5 in China: occurrence in multiple sample types and implications for wild and captive population surveillance. Viruses. 2022;14:411. 35216004 10.3390/v14020411 PMC8875873

[11] Kochagul V, Srivorakul S, Boonsri K, Somgird C, Sthitmatee N, Thitaram C, Pringproa K. Production of antibody against elephant endotheliotropic herpesvirus (EEHV) unveils tissue tropisms and routes of viral transmission in EEHV-infected Asian elephants. Sci Rep. 2018;8:4675. 29549315 10.1038/s41598-018-22968-5 PMC5856810

[12] Hardman K, Dastjerdi A, Gurrala R, Routh A, Banks M, Steinbach F, Bouts T. Detection of elephant endotheliotropic herpesvirus type 1 in asymptomatic elephants using TaqMan real-time PCR. Vet Rec. 2012;170:205. 22186378 10.1136/vr.100270

[13] Bolger AM, Lohse M, Usadel B. Trimmomatic: a flexible trimmer for Illumina sequence data. Bioinformatics. 2014;30:2114–20. 24695404 10.1093/bioinformatics/btu170 PMC4103590

[14] Li H, Durbin R. Fast and accurate short read alignment with Burrows-Wheeler transform. Bioinformatics. 2009;25:1754–60. 19451168 10.1093/bioinformatics/btp324 PMC2705234

[15] Danecek P, Bonfield JK, Liddle J, Marshall J, Ohan V, Pollard MO, Whitwham A, Keane T, McCarthy SA, Davies RM, Li H. Twelve years of SAMtools and BCFtools. Gigascience. 2021;10:giab008. 33590861 10.1093/gigascience/giab008 PMC7931819

[16] Thorvaldsdottir H, Robinson JT, Mesirov JP. Integrative Genomics Viewer (IGV): high-performance genomics data visualization and exploration. Brief Bioinform. 2013;14:178–92. 22517427 10.1093/bib/bbs017 PMC3603213

[17] Garrison E, Marth, G.: Haplotype-based variant detection from short-read sequencing**.** In *arXiv:1207.3907* 2012.

[18] Jung J, Kim JI, Yi G. geneCo: a visualized comparative genomic method to analyze multiple genome structures. Bioinformatics. 2019;35:5303–5. 31350879 10.1093/bioinformatics/btz596 PMC6954651

[19] Saitou N, Nei M. The neighbor-joining method: a new method for reconstructing phylogenetic trees. Mol Biol Evol. 1987;4:406–25. 3447015 10.1093/oxfordjournals.molbev.a040454

[20] Tamura K, Nei M, Kumar S. Prospects for inferring very large phylogenies by using the neighbor-joining method. Proc Natl Acad Sci U S A. 2004;101:11030–5. 15258291 10.1073/pnas.0404206101 PMC491989

[21] Thomas H, Jukes CRC. Evolution of protein molecules. In: Munro HN, editor. Mammalian protein metabolism, vol. 3. Cambridge: Academic Press; 1969. p. 21–132.

[22] Felsenstein J. Confidence limits on phylogenies: an approach using the bootstrap. Evolution. 1985;39:783–91. 28561359 10.1111/j.1558-5646.1985.tb00420.x

[23] Jones DT, Taylor WR, Thornton JM. The rapid generation of mutation data matrices from protein sequences. Comput Appl Biosci. 1992;8:275–82. 1633570 10.1093/bioinformatics/8.3.275

[24] Tamura K, Stecher G, Kumar S. MEGA11: molecular evolutionary genetics analysis version 11. Mol Biol Evol. 2021;38:3022–7. 33892491 10.1093/molbev/msab120 PMC8233496

[25] Stecher G, Tamura K, Kumar S. Molecular evolutionary genetics analysis (MEGA) for macOS. Mol Biol Evol. 2020;37:1237–9. 31904846 10.1093/molbev/msz312 PMC7086165

[26] Gasteiger E, Gattiker A, Hoogland C, Ivanyi I, Appel RD, Bairoch A. ExPASy: the proteomics server for in-depth protein knowledge and analysis. Nucleic Acids Res. 2003;31:3784–8. 12824418 10.1093/nar/gkg563 PMC168970

[27] Richman LK, Zong JC, Latimer EM, Lock J, Fleischer RC, Heaggans SY, Hayward GS. Elephant endotheliotropic herpesviruses EEHV1A, EEHV1B, and EEHV2 from cases of hemorrhagic disease are highly diverged from other mammalian herpesviruses and may form a new subfamily. J Virol. 2014;88:13523–46. 25231303 10.1128/JVI.01673-14 PMC4248956

[28] Barman NN, Choudhury B, Kumar V, Koul M, Gogoi SM, Khatoon E, Chakroborty A, Basumatary P, Barua B, Rahman T, et al. Incidence of elephant endotheliotropic herpesvirus in Asian elephants in India. Vet Microbiol. 2017;208:159–63. 28888631 10.1016/j.vetmic.2017.08.001

[29] Seilern-Moy K, Darpel K, Steinbach F, Dastjerdi A. Distribution and load of elephant endotheliotropic herpesviruses in tissues from associated fatalities of Asian elephants. Virus Res. 2016;220:91–6. 27102836 10.1016/j.virusres.2016.04.012

[30] Holzerlandt R, Orengo C, Kellam P, Alba MM. Identification of new herpesvirus gene homologs in the human genome. Genome Res. 2002;12:1739–48. 12421761 10.1101/gr.334302 PMC187546

[31] Raftery M, Muller A, Schonrich G. Herpesvirus homologues of cellular genes. Virus Genes. 2000;21:65–75. 11022790

[32] Azab W, Osterrieder K. Initial contact: the first steps in herpesvirus entry. Adv Anat Embryol Cell Biol. 2017;223:1–27. 28528437 10.1007/978-3-319-53168-7_1

[33] Fayette MA, Minich DJ, Sylvester H, Latimer E. First detection of clinical disease due to elephant endotheliotropic herpesvirus 7a in two african elephants (Loxodonta Africana) in human care. J Zoo Wildl Med. 2024;55:290–4. 38453514 10.1638/2023-0034

