# Supplementary figures and images for: Fatal infection caused by a genetically distinct elephant endotheliotropic herpesvirus type 5 in a captive Asian elephant in Germany
Source: Virol J. 2024 Sep 16;21:221. doi: 10.1186/s12985-024-02477-w (PMC11406788; doi:10.1186/s12985-024-02477-w)

1

2

3

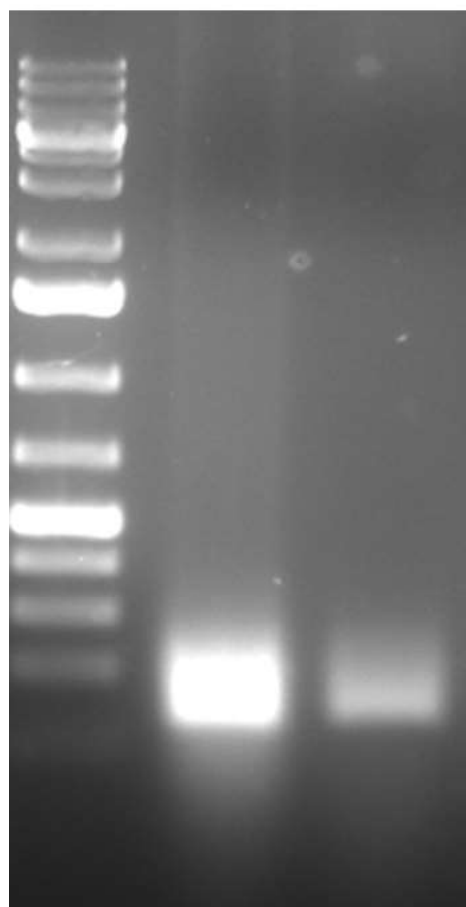

Supplement: Supplementary file 1 — Additional Figure 1: EEHV5 qPCR products visualized by agarose electrophoresis and ethidium bromide staining. Lane 1: 1 kb DNA ladder Plus (Thermo), Lane 2: EEHV5 (Raj spleen), Lane 3: EEHV5 (Raj heart). [file 12985_2024_2477_MOESM1_ESM.pdf]

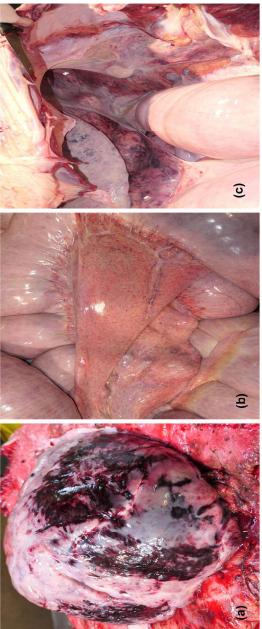

Supplement: Supplementary file 2 — Additional Figure 2: Post-mortem examination of elephant endotheliotropic herpesvirus (EEHV5)-infected elephant. Severe subepicardial petechial hemorrhages and edema in the area of adipose tissue in heart (a). Petechial hemorrhages in serosal surface of large bowel (b) and in abdomen (c). [file 12985_2024_2477_MOESM2_ESM.pdf]

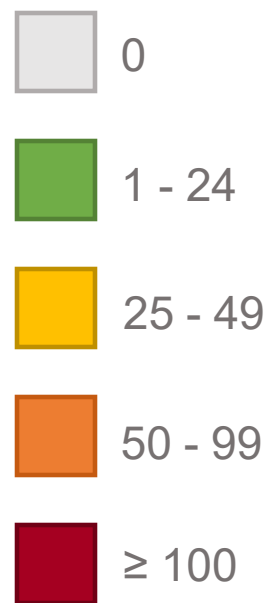

10kbp

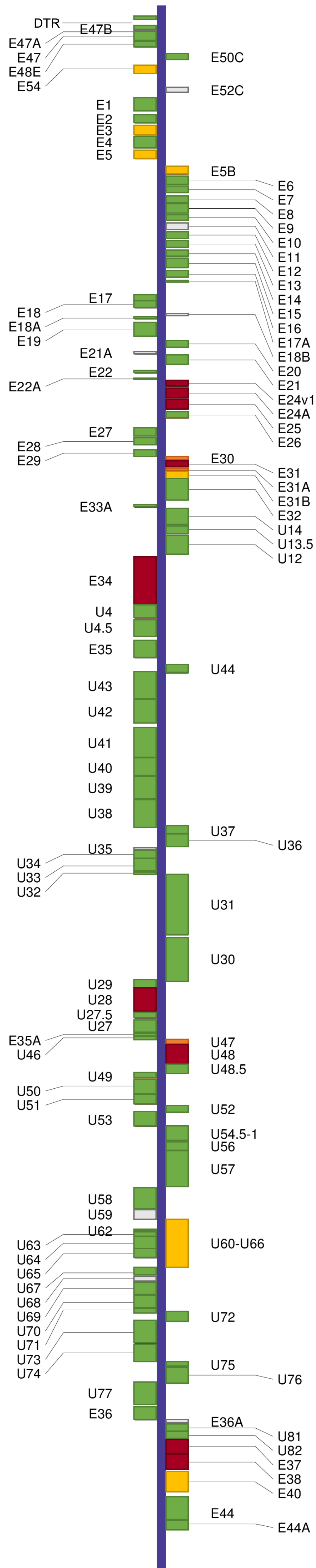

Supplement: Supplementary file 4 — Additional Figure 4: Representation of variant distribution across the EEHV5B Tucker genomic backbone. Color scale on the left illustrates the number of variants present in each gene according to gene color in the map; light blue bar represents the scale, using 10,000 bp (10 Kbp) as a unit. The initial EEHV5B Tucker genome map was drawn using GeneCo and modified to include variant information. [file 12985_2024_2477_MOESM4_ESM.pdf]

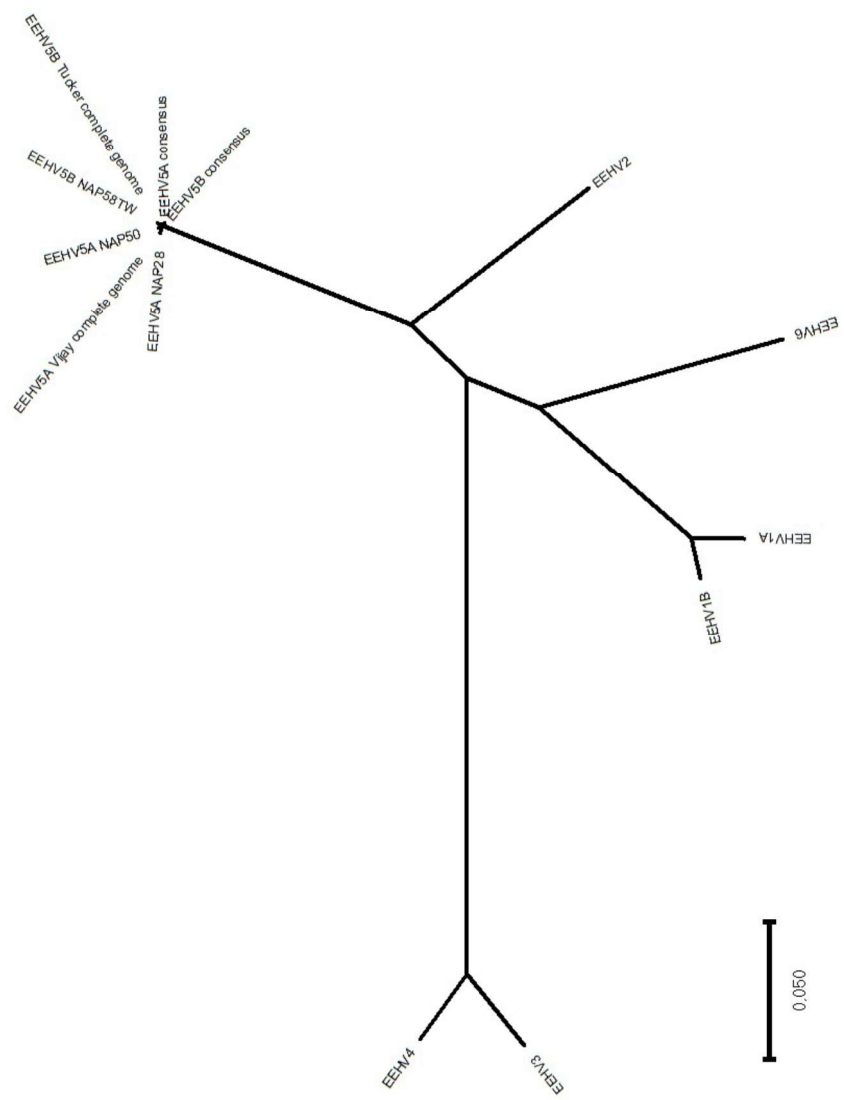

Supplement: Supplementary file 5 — Additional Figure 5: DNA level evolutionary relationships between the EEHV5A and EEHV5B consensus and EEHV family representatives. The radial phylogenetic tree is based on the U38 (POL) gene and was inferred using the Neighbor-Joining method. It includes the EEHV5B Tucker reference genome. All codon positions were included. Ambiguous positions were removed for each sequence pair, leaving 1075 positions in the final dataset. The bar displays the number of nucleotide substitutions per site. Evolutionary analysis was conducted using MEGA11. [file 12985_2024_2477_MOESM5_ESM.pdf]

# U73(OBP)

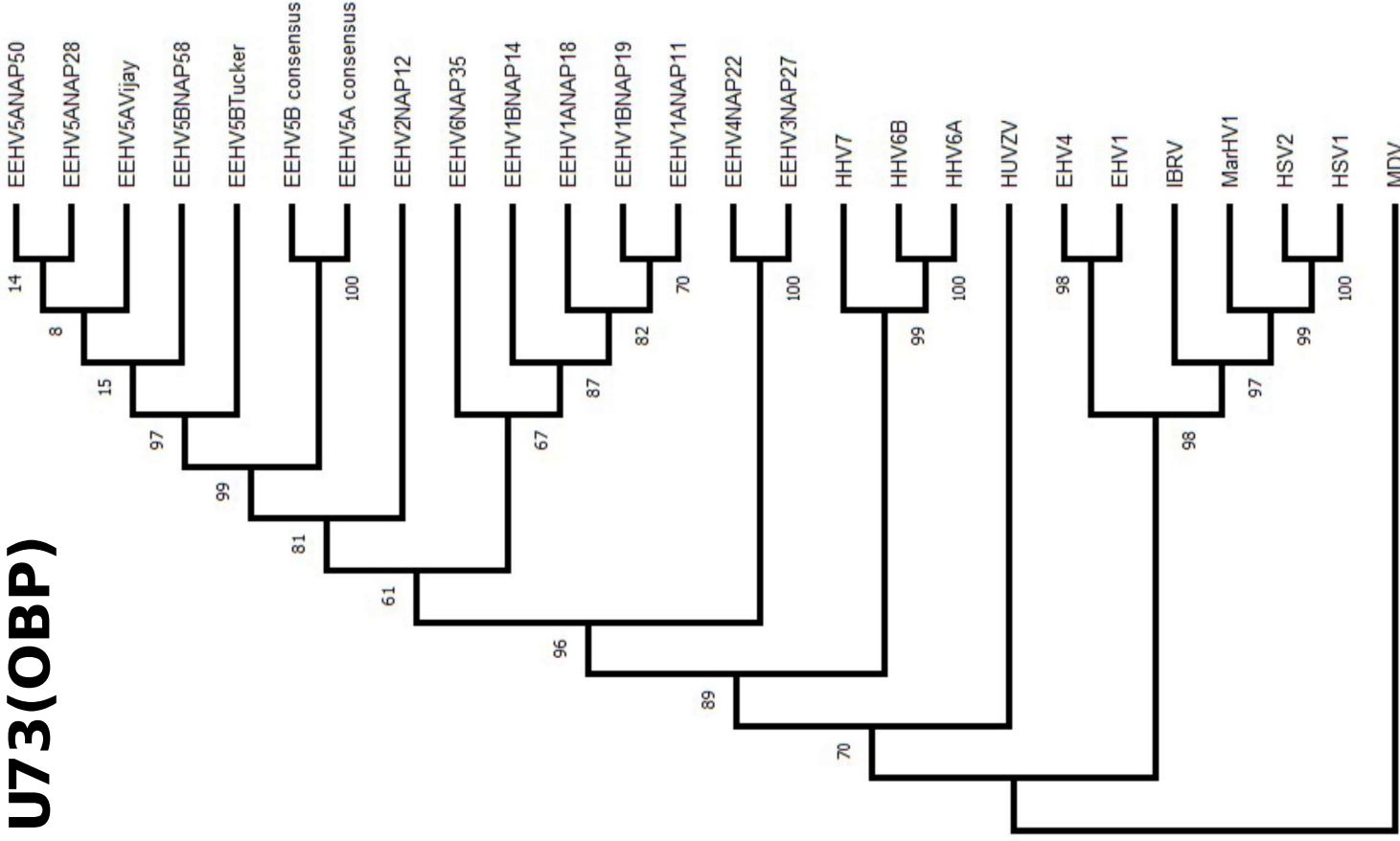

Supplement: Supplementary file 6 — Additional Figure 6: DNA level evolutionary relationships between the EEHV5A and EEHV5B consensus, EEHV family representatives and relevant herpesviruses. The linear phylogenetic trees are based on U38 (POL) and U73 (OBP) genes, respectively. Trees were inferred using the Maximum Likelihood method and Jukes-Cantor model. It includes the EEHV5B Tucker reference genome. All codon positions were included and ambiguous positions were removed for each sequence pair, leaving 1075 positions in the final dataset for U38 (POL) and 672 positions in the final dataset for U73 (OBP). The percentage of replicate trees in which the associated taxa clustered together in the bootstrap test (500 replicates) are shown next to the branches. Evolutionary analysis was conducted using MEGA11. [file 12985_2024_2477_MOESM6_ESM.pdf]

# U81(UDG)

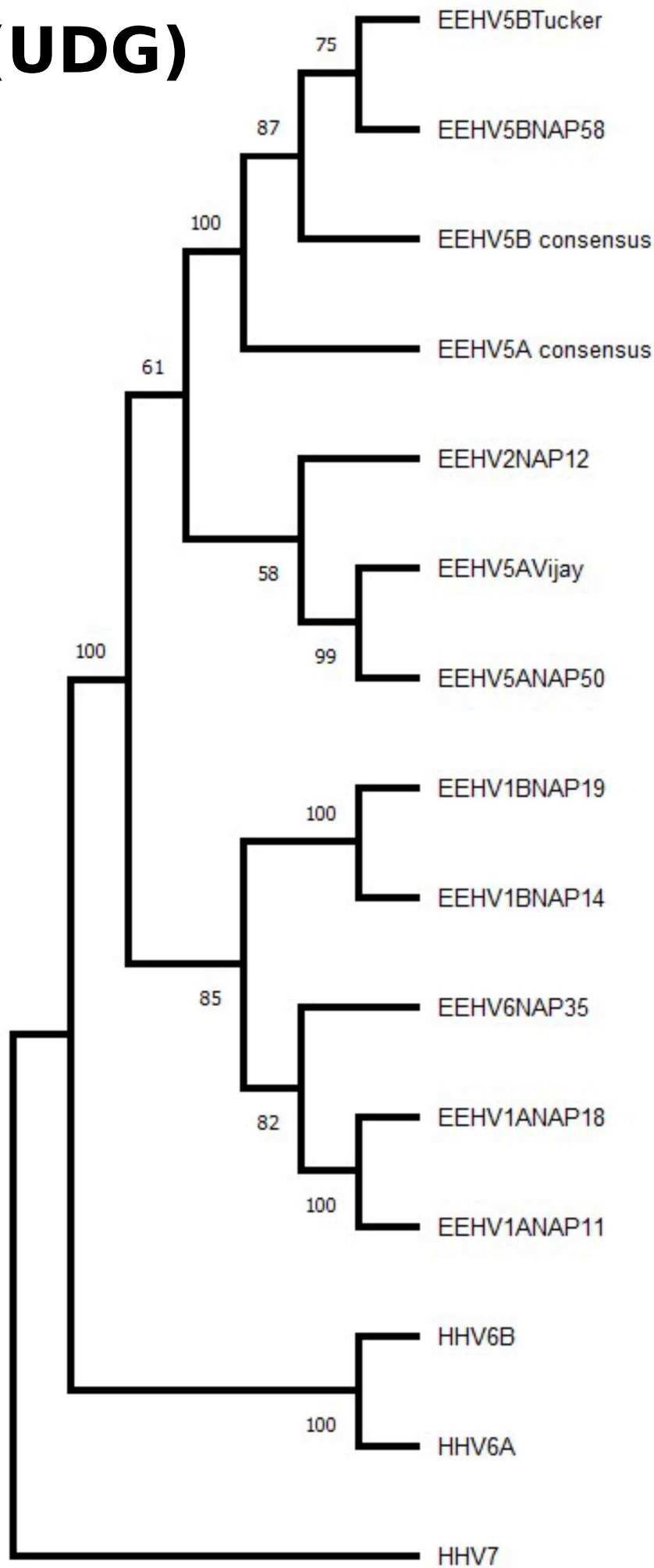

Supplement: Supplementary file 7 — Additional Figure 7: Protein level evolutionary relationships between the EEHV5A and EEHV5B consensus, EEHV and Betaherpesvirinae subfamily representatives. The linear phylogenetic tree is based on the U81 (UDG) protein sequence and was inferred using the Maximum Likelihood method and JTT matrix-based model. It includes the EEHV5B Tucker reference genome. The final dataset included 257 positions. The percentage of trees in which the associated taxa clustered together is shown below the branches. Full evolutionary analysis was conducted using MEGA11. [file 12985_2024_2477_MOESM7_ESM.pdf]
