# Supplementary material for: Fatal infection caused by a genetically distinct elephant endotheliotropic herpesvirus type 5 in a captive Asian elephant in Germany
Source: Virol J. 2024 Sep 16;21:221. doi: 10.1186/s12985-024-02477-w (PMC11406788; doi:10.1186/s12985-024-02477-w)
Supplement: Supplementary file 3 — Additional Figure 3: Comparison between the EEHV5A isolate Vijay and EEHV5B isolate Tucker genome maps. Every ORF is indicated by a grey box; the light blue bar represents the scale, using 10,000 bp (10 Kbp) as a unit. The genome map comparison was drawn using GeneCo. [file 12985_2024_2477_MOESM3_ESM.pdf]

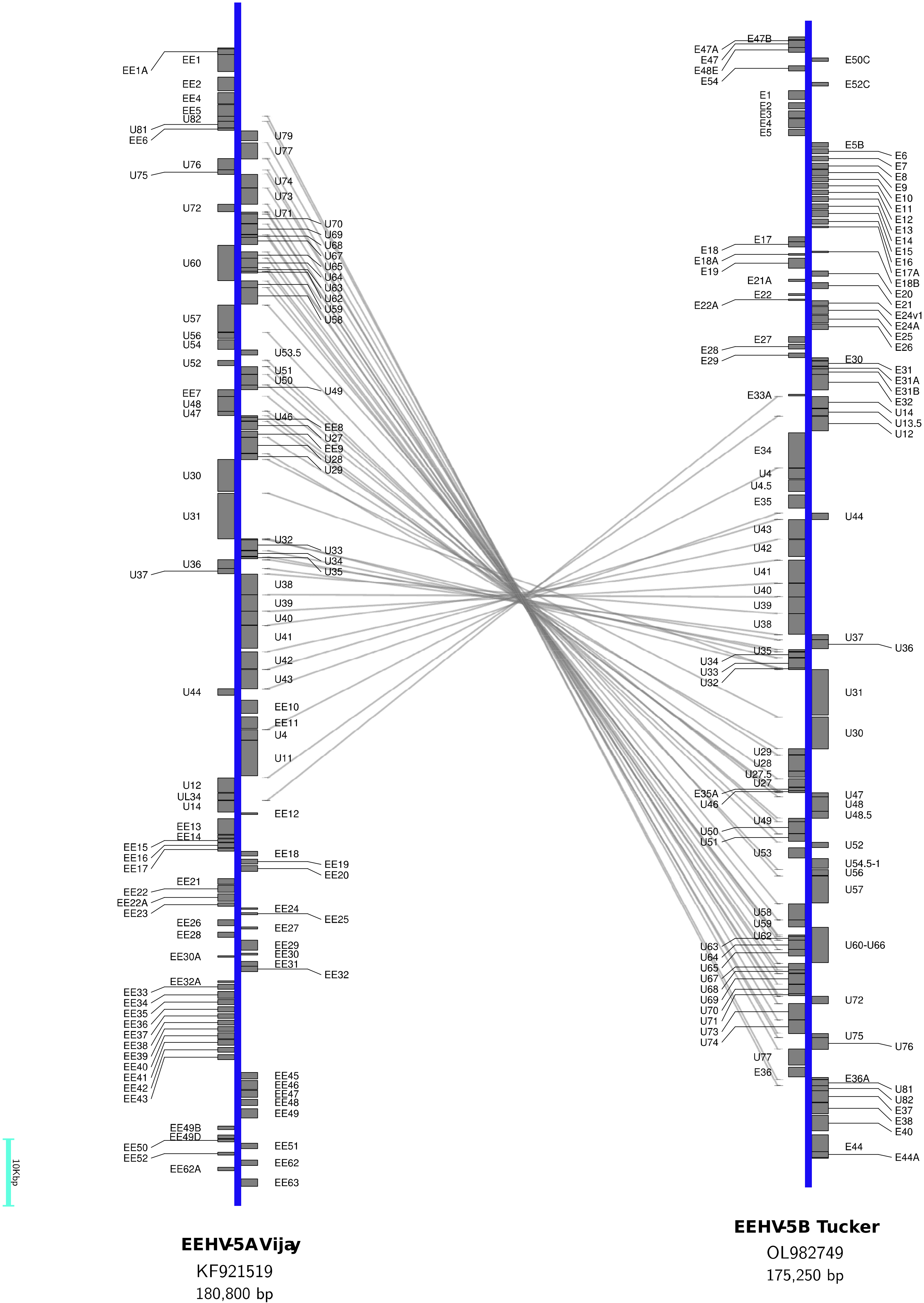
EE1A
EE1
EE2
EE4
EE5
U82
U81
EE6
U79
U77
U76
U75
U74
U73
U72
U71
U70
U69
U68
U67
U65
U64
U63
U62
U59
U58
U60
U57
U56
U54
U53.5
U52
U51
U50
U49
EE7
U48
U47
U46
EE8
U27
EE9
U28
U29
U30
U31
U32
U33
U34
U35
U36
U37
U38
U39
U40
U41
U42
U43
U44
EE10
EE11
U4
U11
U12
UL34
U14
EE12
EE13
EE14
EE15
EE16
EE17
EE18
EE19
EE20
EE21
EE22
EE22A
EE23
EE24
EE25
EE26
EE27
EE28
EE29
EE30
EE30A
EE31
EE32
EE32A
EE33
EE34
EE35
EE36
EE37
EE38
EE39
EE40
EE41
EE42
EE43
EE45
EE46
EE47
EE48
EE49
EE49B
EE49D
EE50
EE51
EE52
EE62
EE62A
EE63
10Kbp
EEHV-5A Vijay
KF921519
180,800 bp
E47B
E47A
E47
E48E
E54
E50C
E52C
E1
E2
E3
E4
E5
E5B
E6
E7
E8
E9
E10
E11
E12
E13
E14
E15
E16
E17
E18
E18A
E19
E17A
E18B
E20
E21A
E22
E22A
E21
E24v1
E24A
E25
E26
E27
E28
E29
E30
E31
E31A
E31B
E32
U14
U13.5
U12
E33A
E34
U4
U4.5
E35
U44
U43
U42
U41
U40
U39
U38
U37
U36
U35
U34
U33
U32
U31
U30
U29
U28
U27.5
U27
E35A
U46
U47
U48
U48.5
U49
U50
U51
U52
U53
U54.5-1
U56
U57
U58
U59
U62
U60-U66
U63
U64
U65
U67
U68
U69
U70
U71
U73
U74
U72
U75
U76
U77
E36
E36A
U81
U82
E37
E38
E40
E44
E44A
EEHV-5B Tucker
OL982749
175,250 bp
